# Fit with good fat? The role of n-3 polyunsaturated fatty acids on exercise performance

**DOI:** 10.1016/j.metabol.2016.10.007

**Published:** 2017-01

**Authors:** Mariasole Da Boit, Angus M. Hunter, Stuart R. Gray

**Affiliations:** a Department of Life and Natural Sciences, University of Derby, Derby, England, DE22 1GB, UK; b Health & Exercise Sciences Research Group, School of Sport, University of Stirling, Stirling, Scotland, FK9 4LA, UK; c Institute of Cardiovascular and Medical Sciences, University of Glasgow, Glasgow, G12 8QQ, UK

**Keywords:** Athletes, Exercise performance, Fish oil, Nutrition, ALA, Alpha-linolenic acid, CVD, Cardiovascular disease, DHA, Docosahexaenoic acid, DOMS, Delayed onset muscle soreness, DPA, Docosapentaenoic acid, EIB, Exercise-induced bronchoconstriction, EPA, Eicosapentaenoic acid, FA, Fatty acid, HRV, Heart rate variability, MPS, Muscle protein synthesis, n-3 PUFA, (n-3) Polyunsaturated fatty acids, PBMC, Peripheral blood mononuclear cells, ROS, Reactive oxygen species, TBARS, Thiobarbituric acid reacting substances, URTI, Upper respiratory tract infection

## Abstract

N-3 PUFA (n-3) polyunsaturated fatty acids (PUFA) are a family of fatty acids mainly found in oily fish and fish oil supplements. The effects of n-3 PUFA on health are mainly derived from its anti-inflammatory proprieties and its influence on immune function. Lately an increased interest in n-3 PUFA supplementation has reached the world of sport nutrition, where the majority of athletes rely on nutrition strategies to improve their training and performance. A vast amount of attention is paid in increasing metabolic capacity, delaying the onset of fatigue, and improving muscle hypertrophy and neuromuscular function. Nutritional strategies are also frequently considered for enhancing recovery, improving immune function and decreasing oxidative stress. The current review of the literature shows that data regarding the effects of n-3PUFA supplementation are conflicting and we conclude that there is, therefore, not enough evidence supporting a beneficial role on the aforementioned aspects of exercise performance.

## 1 Introduction

There has always been an interest in using different nutrients and supplements for improving athletic performance and recovery. Indeed, many athletes use daily dietary supplements with most attention usually on increasing metabolic capacity, delaying the onset of fatigue, improving muscle hypertrophy, and shortening recovery periods. Moreover, exercise training and competition are physiologically demanding for athletes and as such they can face a temporary reduction in immune function, where they are more at risk of upper respiratory tract infections (URTI). Athletes are, therefore, constantly seeking effective nutrients to maintain good health and avoid disruptions to their training regime. N-3 PUFA (n-3) polyunsaturated fatty acids (PUFA) have recently been considered as a supplement which may have a role in the above processes [1], although the evidence for such claim is still premature. This review will consider different aspects of exercise performance and trials in this area using n-3 PUFA supplementation.

The n-3 PUFA family, derived from the essential fatty acid alpha-linolenic acid (ALA), is involved in important body functions. In the modern Western diet n-3 PUFA can be found in botanical sources, which are rich in ALA; and in marine sources, e.g. oily fish (e.g. salmon), crustacean (e.g. krill) and the liver of lean fish, which are rich in eicosapentaenoic acid (EPA), docosahexaenoic acid (DHA) and docosapentaenoic acid (DPA). These n-3 PUFA are also frequently called ‘essential’ fatty acids, as they cannot be readily synthesized de novo by the body. Hence, it is important to consume n-3 PUFA to provide our body with the amount of EPA and DHA necessary for optimal physiological functioning [2].

The effects of n-3 PUFA on health are mainly derived from its immunomodulatory and anti-inflammatory proprieties and its influence on immune function [3]. Through these properties it has been demonstrated that n-3 PUFA supplementation may help in the prevention or treatment of many inflammatory-related diseases such as diabetes [4], [5], [6] and cardiovascular disease [7], [8], [9], [10], [11], [12], [13]. However, in recent years, the certainties of this assertion have been challenged by new discoveries [14], [15]. Particularly, recent meta-analysis has failed to prove any associations between n-3 PUFA supplementation and improvements in vascular diseases [16], [17] or diabetes [18].

Because of the aforementioned properties over the last decade or so there has also been an increasing interest in potential benefits of n-3 PUFA supplementation in athletic populations, with an ultimate goal to improve athletic/sporting performance. The aim of the current review is to give a comprehensive overview of the current literature which has investigated the effect of n-3 PUFA supplementation in athletic/sporting performance (Table 1).

## 2 Skeletal Muscle

Good muscle health is one of the main aspects influencing athletes' performance. Skeletal muscle performance is frequently determined by the use of standard measurements such as rate of muscle protein synthesis (MPS), muscle mass, maximum voluntary contraction, rate of torque development, and for recovery measures of soreness, swelling and markers of muscle damage. Regarding the maintenance and hypertrophy of muscle, research has highlighted positive effects of n-3 PUFA on muscle anabolism and catabolism [19], [20], [21]. Although early research focused mainly on cancer cachexia [22], [23], more recent studies suggest a positive impact also in healthy people. Indeed, Smith et al. [19] showed that 8 weeks of n-3 PUFA (4 g/day) administration significantly increased (*p* < 0.05) MPS in young and older healthy subjects during a hyperaminoacidemic–hyperinsulinemic clamp. These findings are also supported by animal [20], [24], as well as in vitro studies [25], [26]. While of clear importance, these short-term studies do not necessarily mean that long-term increases in muscle mass and function will be observed with n-3 PUFA supplementation.

However, in recent work 6 months of supplementation with 3.36 g of n-3 PUFA (1.86 g EPA and 1.50 g DHA) daily resulted in a significant increase (*p* < 0.05) in thigh muscle volume (of 3.6%) and muscle strength (of 4.0%) in older people [21]. Furthermore, when n-3 PUFA is combined with physical activity similar effects have been noted [27]. In this study 90 days of strength training improved peak torque and rate of torque development to a greater extent (*p* < 0.05) in older women, supplemented with 2 g/day of n-3 PUFA (~ 0.4 g EPA and 0.3 g DHA/day) for 90 or 150 days, compared to the group receiving only the training. Interestingly, our recent work has indicated that this effect may only be seen in older women but not older men (unpublished data). Whilst these data in older people are of interest and important, its relevance to young athletic populations undertaking regular strength training, where the anabolic response to exercise alone will be closer to maximal compared to older people, is not clear. Indeed, recent work has shown that 8 weeks of supplementation with 4.5 g/day n-3 PUFA (3.5 g EPA, 0.9 g DHA and 0.1 g DPA) in young healthy males had no effect on MPS measured after a bout of resistance exercise and the ingestion of 30 g whey protein [28]. The long-term effect of n-3 PUFA supplementation in such populations remains to be established.

Muscle recovery after an exercise bout might influence training adaptations. In recent years considerable interest has been shown in the effect of n-3 PUFA on muscle recovery, damage and soreness – although the findings are mixed. In one study eleven healthy adults performed eccentric biceps curls at 120% of their 1RM after 14 days of dietary n-3 PUFA restriction and again after 7 days of 3 g/day n-3 PUFA supplementation. The results showed that n-3 PUFA supplementation decreased (*p* < 0.05) post-exercise soreness [29], with similar findings reported in other studies [30], [31], [32], [33]. Further studies support these findings with n-3 PUFA attenuating (*p* < 0.05) blood markers of muscle damage (skeletal muscle slow troponin I, myoglobin, creatine kinase), inflammation (TNF-α), DOMS and loss of strength and range of motion [34]. On the other hand Lenn et al. [35] did not find any change in strength, pain or markers of muscle damage and inflammation following 50 maximal effort isokinetic eccentric elbow flexion contractions after 30 days of 1.8 g/day of n-3 PUFA in healthy volunteers. Similarly, 6-week supplementation with 3 g/day n-3 PUFA did not alter force recovery, muscle soreness or markers of muscle damage after eccentric contractions of the knee extensor muscles in recreationally active individuals (age 23 ± 2.3, mean ± SD) [36]. It is, therefore, unclear whether n-3 PUFA can be recommended to athletic populations to improve these aspects of recovery during training or in competition. A possible explanation for these contrasting results is the use of different types of exercise to induce muscle damage, as well as dose and duration of n-3 PUFA supplementations among studies.

## 3 Energy Metabolism

The availability of energy, i.e. the supply of ATP to the actin-myosin cross-bridge, is clearly another important aspect of exercise performance. To date the information available regarding energy metabolism and n-3 PUFA in humans is very limited, while there are many in vitro and animal studies [37], [38], [39]. However, in this review we have chosen to focus on human studies.

Healthy skeletal muscle tissue is characterized by metabolic flexibility, which is the ability to switch from one substrate to another when required. Briefly, metabolic flexibility in human muscle cells comprises suppressibility: the ability of glucose to suppress fatty acid (FA) oxidation; adaptability: the ability of cells to increase FA oxidation upon increased FA availability; and substrate-regulated flexibility: the ability to increase FA oxidation when changing from a high glucose, low fatty acid condition to a high fatty acid, low glucose condition. In an in vitro study Hessvik and colleagues found that 24 hours of exposure with 100 μM EPA in human myotubes significantly increased suppressibility, adaptability, substrate-regulated flexibility and upregulated specific genes involved in fatty acid β-oxidation compared to pre-treatment with unsaturated fatty acid oleic acid [40]. This suggests an overall improvement in skeletal muscle metabolic flexibility after EPA administration. In agreement with these findings a recent metabolic study reported that treatment with n-3 PUFA prevents metabolic dysfunction in skeletal muscle of mice, by limiting the accumulation of intramyocellular lipid in type I muscle fibres [41]. According to these findings n-3 PUFA supplementation might be beneficial for endurance athletes who rely on fatty acid as substrate to sustain prolonged efforts.

Nevertheless, n-3 PUFA seems to have little effect on human metabolism [42]. Six weeks of n-3 PUFA (2.4 g/day) supplementation failed to modify both resting metabolic rate (n-3 PUFA = + 17 ± 260 kcal, placebo = − 62 ± 184 kcal; *p* > 0.05) and respiratory exchange ratio (n-3 PUFA = − 0.02 ± 0.09, placebo = + 0.02 ± 0.05; *p* > 0.05) in healthy subjects. Additionally, Bortolotti et al. [43] investigated whether 14 days of 7.2 g/day n-3 PUFA supplementation affect energy and substrate metabolism during a 30 min cycling exercise at 50% VO_2max_. They reported neither changes in energy expenditure derived from carbohydrate oxidation (83 ± 2% vs 84 ± 1%, *p* > 0.05) nor from fat oxidation (13 ± 1% vs 11 ± 1%, *p* > 0.05) in control conditions and after n-3 PUFA supplementation, respectively. Moreover, there was no difference in VO_2max_ (38.4 ± 2.0 mL kg^− 1^ min^− 1^ vs 38.6 ± 2.2 mL kg^− 1^ min^− 1^, *p* > 0.05) between the 2 groups.

It is evident that, with only a few contradictory studies available, drawing any conclusion on the effect of n-3 PUFA on energy metabolism would be inappropriate.

## 4 Endurance Performance

There have been several studies investigating the effects of n-3 PUFA supplementation on cardiovascular responses to a bout of endurance exercise. In myocardial infarction survivors, supplementation with 810 mg/day of DHA and EPA, versus corn/olive oil placebo, for 4 months reduced resting HR with concomitant increases in stroke volume, cardiac ejection time, heart rate variability (HRV) and HR recovery after exercise [44]. In the same year Walser and colleagues [45] investigated the effects of 6 weeks of supplementation with 500 mg/day DHA and EPA, versus safflower placebo, in healthy volunteers. In this study no changes in blood pressure or HR, both at rest and during exercise, were observed. Resting brachial artery diameter, conductance and blood flow were not altered by n-3 PUFA supplementation but the increases in these measures, during contraction, were greater with the supplementation.

More recent studies, in young healthy people, have generally confirmed that n-3 PUFA supplementation can alter various aspects of the cardiovascular system. N-3 PUFA supplementation has been found to lower submaximal and peak HR, and whole body oxygen consumption during exercise [46], reduce resting and submaximal HR and increase high-frequency power for HRV [47], reduce diastolic BP and submaximal HR [48], decrease systemic vascular resistance [49], reduce oxygen consumption and RPE [50] and reduce resting HRV and submaximal heart rate [51]. Furthermore in in vivo studies, in rat hindlimb muscle, n-3 PUFA supplementation reduced skeletal muscle oxygen consumption during contractions [52]. It is worth pointing out that in our own work we have been unable to find any differences in HR or oxygen consumption either a rest or during submaximal exercise with either fish or krill oil supplementation [53], [54].

Whilst the majority of these studies show beneficial cardiovascular effects of n-3 PUFA supplementation during exercise, whether these result in any increases in exercise performance is far from guaranteed. In fact, looking at the literature it is evident that n-3 PUFA supplementation has no effect on endurance exercise performance. For instance, no differences in VO_2max_, maximal power or endurance performance (time to completion) were found in healthy well-trained cyclists after 3 weeks of n-3 PUFA (6 g/day), n-3 PUFA + vitamin E (300 IU) or placebo (microcrystalline cellulose) [55]. These findings have been supported by recent work demonstrating no effect of n-3 PUFA supplementation on endurance performance or recovery in Australian Rules footballers (treadmill run to exhaustion) [48], well-trained cyclists (cycle to exhaustion) [46] or young healthy people (cycling simulated time trial) [54]. On the other hand, one study did find that n-3 PUFA supplementation (1.1 g/day), versus placebo (lactose monohydrate), resulted in a 3.7 mL kg^− 1^ min^− 1^ increase in VO_2max_, alongside an increase in endothelial function, although no endurance performance measure was made [56]. Overall, while n-3 PUFA supplementation confers some “beneficial” effects on cardiovascular function, during exercise, this does not translate into an improvement in endurance performance.

## 5 Immune Function

Over the last ~ 30 years many studies have investigated the effects of exercise on the function of the immune system (see [57]). In brief, those participating in high levels of endurance exercise are more susceptible to the development of URTI [58], and this can interfere with performance during training and in competition. Furthermore, after a single bout of high-intensity long-duration endurance exercise there is clear evidence of an immunosuppression [59], [60], [61], [62], [63], [64]. Based on previous work that n-3 PUFA supplementation can alter the function of the immune system, it has been hypothesised that such supplementation could modulate immune function after exercise and potentially reduce the incidence of URTI.

Research investigating the effects of n-3 PUFA supplementation on immune function and exercise has, however, shown mixed results. In the first such study it was shown that n-3 PUFA supplementation (3.6 g/day), in young endurance-trained runners, for 6 weeks did not alter plasma cytokine levels, creatine kinase levels or immune cell numbers [65], with similar findings reported by Nieman and colleagues [66]. In further research in elite competitive swimmers it has been shown that n-3 PUFA supplementation (1.8 g/day), versus placebo (mineral oil), for 6 weeks in the run up to a major competition reduces prostaglandin E2 levels, and increases PBMC proliferation and the production of IFN-γ [67]. In our recent work we have found that n-3 PUFA supplementation (1.6 g/day), versus placebo (olive oil), for 6 weeks in young healthy males, results in an increase in PBMC IL-2 production and NK cell cytotoxic activity 3 h after a single bout of endurance exercise [50]. Other measures that were found not to change with n-3 PUFA supplementation were neutrophil phagocytosis and oxidative burst, plasma IL-6 and cortisol, and PBMC IL-4 and IFN-γ production. Interestingly, we have also found similar results in our recent study employing krill oil (360 mg/day), versus placebo (oil mix similar to average European diet), when investigating young healthy individuals [54]. Summarising, it appears that n-3 PUFA does have some effect in modulating the immune system in the recovery period after exercise but whether it can reduce the incidence of URTI remains to be established.

In a recent study we investigated the effects of a daily supplement drink containing 1.1 g n-3 PUFA, 8 g whey protein and 10 μg vitamin D3, versus placebo drink, for 16 weeks, in the incidence of URTI in a young active population. The daily supplement resulted in a reduction in the number URTI symptom days, although the actual number of URTI episodes and severity of each URTI did not differ between supplemented and placebo groups, nor did the concentration and secretion rate of IgA in saliva samples [68]. Whether this small effect on URTI symptom days is due to the n-3 PUFA in the supplement is not clear and so further work is needed to establish if n-3 PUFA supplementation can reduce URTI incidence in athletic populations.

N-3 PUFA have been shown to benefit people with exercise-induced bronchoconstriction (EIB) [69]. While the mechanisms underlying EIB are not fully uncovered, there is strong evidence that airway inflammation is involved [70]. Many elite athletes suffer from EIB and this can be a limiting factor in their performance [71]. Strategies to reduce the effects of EIB were, therefore, clearly warranted. The group of Mickleborough and colleagues [72] have investigated the effects of n-3 PUFA supplementation (5.2 g/day), versus placebo (olive oil), in asthmatics with documented EIB on exercise-induced airway narrowing and inflammation. In this study n-3 PUFA supplementation resulted in a marked improvement in post-exercise lung function and reduced concentrations of sputum immune cell (eosinophil and neutrophil) count, pro-inflammatory eicosanoid (LTC_4_-LTE_4_, and PGD_2_) concentrations and cytokine (IL-1β and TNF-α) concentrations. So, while it is unclear whether athletic populations in general would benefit from n-3 PUFA supplementation to improve immune function, there may be a potential beneficial effect for those suffering from EIB, although higher doses of n-3 PUFA were employed in this specific population.

## 6 Oxidative Stress

It is possible that due to the high number of double bonds present an increase in n-3 PUFA fatty acid intake may lead to an increase in lipid peroxidation and the generation of a state of oxidative stress [73], which has been linked to many disease states such as Parkinson's [74] and cardiovascular disease [75]. However, an optimal level of reactive oxygen species (ROS) production has a positive signalling role [76]. Indeed, there is evidence that this ROS production is important in modulating skeletal muscle contractile function, promoting mitochondrial biogenesis and for normal skeletal muscle remodelling in response to exercise [75]. In fact, supplementation with high levels antioxidants (vitamins C and E) blunts many of these beneficial adaptions [77], although others do not support this finding [78].

The results of previous investigations into the effect of n-3 PUFA supplementation on resting levels of markers of oxidative stress are conflicting. It has been demonstrated that 6 weeks n-3 PUFA supplementation (3.4 g/day), versus placebo (sunflower oil), in postmenopausal women resulted in a 23% increase in thiobarbituric acid reacting substances (TBARS), with no changes in plasma F_2_-isoporstanes [79]. Further evidence of increased oxidative stress has also been found in further studies [80], [81], while other work has shown reductions in markers of oxidative stress with n-3 PUFA supplementation [82], [83], [84].

In exercise studies the results are equally as ambiguous. N-3 PUFA supplementation was found to reduce, post-exercise, the rate of LDL oxidation [55], TBARS and DNA damage [36]. On the other hand McAnulty et al. [85] showed that supplementation with n-3 PUFA (2.4 g/day), versus placebo (soybean oil) in trained cyclists resulted in a greater post-exercise plasma concentration of F2-isoprostanes following three days of 3-hour cycling sessions. In addition, there are studies which find no effect of n-3 PUFA supplementation on exercise induce markers of oxidative stress [86], [87]. A great deal of the confusion in this area likely relates to the many different participant groups, exercise protocols and chosen marker/markers (and analytical techniques) of oxidative stress. However, even when taking these into account, it is not clear to which groups of people participating in what form of exercise n-3 PUFA supplementation may have any effect. It is also not clear what effect, if any, alterations in free radical production or antioxidant capacity would have on adaptations to exercise training, and so recommending n-3 PUFA supplementation on this basis would be futile.

## 7 Neuromuscular Function

As mentioned above, supplementation with n-3 PUFA for 150 days has been reported to enhance strength [27] and also neuromuscular recruitment following exercise training programmes [88]. The main acid thought to be responsible for this is DHA as it is the essential constituent of neuronal membrane phospholipids [88], [89] and thus considered fundamental for development of normal brain function and neuronal pathways [90].

Strong evidence for this role of DHA exists on preterm infants who have lower DHA levels when compared to term infants [91] and as such are typically smaller and neurologically underdeveloped [92]. This is further supported by a series of convincing studies demonstrating enhanced neural and body mass development in preterm infants supplemented with DHA [93], [94], [95], [96], [97]. Although the precise mechanisms for such development is unclear, Lewis et al. [88] demonstrated enhanced neuromuscular development after 21 days of n-3 PUFA supplementation during exercise training and the authors proposed this may have been caused by increased acetylcholine concentration and acetylcholinesterase activity at the neuromuscular junction. However, this theory was derived from an animal study [98] therefore comparing this to dynamics of human skeletal muscle makes the theory quite speculative. Other potential theories include alterations in membrane composition and fluidity [98], which may accelerate conductance of action potentials down the neurons [99] to increase motor unit firing rate onto the sarcolemma; but the same issues exist when attempting to relate this to human skeletal muscle. Nevertheless, it is possible that chronic n-3 PUFA supplementation may enhance neuromuscular activity, via DHA, but more research needs to be carried out to establish this proposed effect, particularly in well-adapted athletic populations.

## 8 Conclusion

Whilst n-3 PUFA supplementation has been recommended for athletic populations on the basis of the available literature [1], at this time, we find no merit in such a recommendation. There are certain populations where n-3 PUFA supplementation might be a potential aid (i.e. athletes with EIB) and it may be the case that other groups would benefit (i.e. strength athletes) but there is currently a paucity of data from high quality studies in this area.

## Conflict of Interest

The authors have no conflict of interest to declare.

## Funding Information

MDB was employed via a grant from the BBSRC (Grant number BB/J015911/1) to SRG.

## Figures and Tables

**Table 1:** Effects of n-3 PUFA supplementation on exercise performance.

Reference (y)	Population (n)	n-3 PUFA dose (g/d)	Exercise	Supplementation duration	Effects of omega 3
Rodacki et al. (2012)	Healthy women, 64 ± 1.4 y (n = 45)	2	90 days strength training	90 and 150 days	Peak torque ↑ Rate of torque development ↑
McGlory et al. (2016)	Healthy men, 20.5 ± 0 y (n = 20)	4.5	Acute bout of resistance exercise	8 weeks	MPS →
Jouris et al. (2011)	Healthy, 35 ± 10 y (n = 11)	3	Acute bout of eccentric biceps curls (120% 1RM)	7 days	DOMS ↓
Lembke et al. (2014)	Healthy, 18.6 ± 1.2 y (n-3 PUFA: n = 43) and 18.9 ± 1.1 y (placebo: n = 22)	2.7	Acute bout of maximum eccentric forearm extensions	30 days	DOMS ↓ Blood lactate ↓ C-reactive protein ↓
Corder et al. (2016)	Healthy women, 33 ± 2 y (n = 27)	3	Maximum eccentric biceps curl exercises	9 days	DOMS ↓ Skin temperature → C-reactive protein →
Tsuchiya et al. (2016)	Healthy men, 19.5 ± 0.8 y (n = 24)	0.86	Maximum eccentric elbow flexion exercises	8 weeks	DOMS ↓ Strength and range of motion ↑ IL-6 ↓
Tinsley et al. (2016)	Healthy women, 22.5 ± 1.8 y (n-3 PUFA: n = 8) and 24.7 ± 3.6 y (placebo: n = 9)	6	10 sets to failure of elbow flexion and leg extension machines	1 week	DOMS ↓
Mickleborough et al. (2015)	Untrained healthy, 22.0 ± 2 y (n = 32)	1.2	Downhill running (− 16% grade)	26 days	Blood markers of muscle damage/inflammation ↓ DOMS ↓ Strength and range of motion ↑
Lenn et al. (2002)	Healthy, 22.7 ± 3.9 y men (n = 13) and 24.5 ± 5.4 y women (n = 9)	1.8	Maximum isokinetic eccentric elbow flexion	30 days	Muscle strength → DOMS → Blood markers of muscle damage/inflammation →
Gray et al. (2014)	Healthy, 23 ± 2.3 y (n = 20)	3	Maximum eccentric knee extensor muscles contractions	6 weeks	Muscle strength → DOMS → Blood markers of muscle damage → TBARS and cellular DNA damage ↓
Bortolotti et al. (2007)	Healthy men, 24 ± 1 y (n = 8)	7.2	30 min cycling exercise (50% VO_2max_)	14 days	Energy metabolism → VO_2max_ →
Peoples et al. (2008)	Well-trained men, 27.1 ± 2.7 y (placebo: n = 7) and 23.2 ± 1.2 y (n-3 PUFA: n = 9)	8	Sustained submaximal exercise tests (55% of peak workload)	8 weeks	Submaximal and peak HR and oxygen consumption during exercise ↓ VO_2peak ,_ time to exhaustion and peak workload →
Ninio et al. (2008)	Overweight, 25–65 y (n = 65)	6	Aerobic exercise (45 min, 3 times a week, at 75% HR_max_)	12 weeks	Resting and submaximal HR during exercise ↓ HRV (high-frequency)↑
Buckley et al. (2009)	Footballers, 21.7 ± 1.0 y (n-3 PUFA) and 23.2 ± 1.1 y (placebo) (n = 25)	6	2 treadmill runs to exhaustion	5 weeks	Diastolic BP and submaximal HR during exercise ↓ VO_2peak ,_ time to exhausition and recovery time →
Rontoyanni et al. (2012)	Healthy men, 18–45 y (n = 22)	4.7	12 min multi-stage exercise stress (25 W increase)	Single dose	Systemic vascular resistance ↓ Cardiac output during exercise →
Kawabata et al. (2014)	Healthy, 23 ± 1 y (n = 20)	3.6	Submaximal exercise test (30 min at 2-mM of BLa, followed by 30 min at 3-mM)	8 weeks	Oxygen consumption and RPE ↓
Macartney et al. (2014)	Healthy, 18–40 y (n = 26)	2	5 min maximum work capacity trial	8 weeks	Resting and submaximal HR and HR recovery ↓ HR peak →
Gray et al. (2012)	Healthy, 24 ± 3.8 y (n = 16)	2	1 h cycling (70% VO_2peak_)	6 weeks	HR and O_2_ consumption, at rest and during submaximal exercise →
Da Boit et al. (2015)	Healthy, 25.8 ± 5.3 y (n = 37)	2	Cycling time trial to fixed energy expenditure	6 weeks	Time trial completion time, HR and O_2_ consumption, at rest and during submaximal exercise →
Oostenbrug et al. (1997)	Trained cyclists, 19–42 y (n = 24)	6	Cycling time trial of 1 h	3 weeks	VO_2max_, maximal power and time to exhaustion →
Żebrowska et al. (2015)	Cyclists, 23.1 ± 5.4 y (n = 13)	1.3	VO_2max_ cycling test	3 weeks	VO_2max_ and endothelial function ↑
